# Use of artificial intelligence in ophthalmology: a narrative review

**DOI:** 10.1590/1516-3180.2021.0713.R1.22022022

**Published:** 2022-08-29

**Authors:** Thiago Gonçalves dos Santos Martins, Paulo Schor, Luís Guilherme Arneiro Mendes, Susan Fowler, Rufino Silva

**Affiliations:** I MD, PhD. Researcher, Department of Ophthalmology, Universidade Federal de São Paulo (UNIFESP), São Paulo (SP), Brazil; Research Fellow, Department of Ophthalmology, Ludwig Maximilians University (LMU), Munich, Germany; and Doctoral Student, University of Coimbra (UC), Coimbra, Portugal.; Ludwig Maximilians University, Department of Ophthalmology, Munich, Germany; University of Coimbra, Coimbra, Portugal; II PhD. Professor, Department of Ophthalmology, Universidade Federal de São Paulo (UNIFESP), São Paulo (SP), Brazil.; III PhD. Engineer, Association for Innovation and Biomedical Research on Light and Image (AIBILI), Coimbra, Portugal.; IV RN, PhD. Certified Neuroscience Registered Nurse (CNRN) and Research Fellow of American Heart Association, Department of Ophthalmology, Orlando Health, Orlando, United States; Researcher, Department of Ophthalmology, Walden University, Minneapolis (MN), United States; and Researcher, Department of Ophthalmology, Thomas Edison State University (TESU), Trenton (NJ), United States.; Walden University, Department of Ophthalmology, Minneapolis, United States; Thomas Edison State University, Department of Ophthalmology, Trenton, NJ, United States; V MD, PhD. Fellow of the European Board of Ophthalmology and Professor, Coimbra Institute for Clinical and Biomedical Research (iCBR), Faculty of Medicine, University of Coimbra, Coimbra, Portugal; Fellow, Department of Ophthalmology, Centro Hospitalar e Universitário de Coimbra (CHUC), Coimbra, Portugal; and Researcher, Association for Innovation and Biomedical Research on Light and Image (AIBILI), Coimbra, Portugal.; Centro Hospitalar e Universitário de Coimbra, Department of Ophthalmology, Coimbra, Portugal; Association for Innovation and Biomedical Research on Light and Image, Coimbra, Portugal

**Keywords:** Artificial intelligence, Glaucoma, Retinopathy of prematurity, Ophthalmology, Neural network, Convolutional neural network, Diabetes, Macular degeneration, Deep reinforcement learning

## Abstract

**BACKGROUND:**

Artificial intelligence (AI) deals with development of algorithms that seek to perceive one’s environment and perform actions that maximize one’s chance of successfully reaching one’s predetermined goals.

**OBJECTIVE:**

To provide an overview of the basic principles of AI and its main studies in the fields of glaucoma, retinopathy of prematurity, age-related macular degeneration and diabetic retinopathy. From this perspective, the limitations and potential challenges that have accompanied the implementation and development of this new technology within ophthalmology are presented.

**DESIGN AND SETTING:**

Narrative review developed by a research group at the Universidade Federal de São Paulo (UNIFESP), São Paulo (SP), Brazil.

**METHODS:**

We searched the literature on the main applications of AI within ophthalmology, using the keywords “artificial intelligence”, “diabetic retinopathy”, “macular degeneration age-related”, “glaucoma” and “retinopathy of prematurity,” covering the period from January 1, 2007, to May 3, 2021. We used the MEDLINE database (via PubMed) and the LILACS database (via Virtual Health Library) to identify relevant articles.

**RESULTS:**

We retrieved 457 references, of which 47 were considered eligible for intensive review and critical analysis.

**CONCLUSION:**

Use of technology, as embodied in AI algorithms, is a way of providing an increasingly accurate service and enhancing scientific research. This forms a source of complement and innovation in relation to the daily skills of ophthalmologists. Thus, AI adds technology to human expertise.

## INTRODUCTION

Early diagnosis of eye diseases is of great importance for preventing loss of vision and thereby improving the quality of life. However, the teaching of ophthalmology has declined in medical schools, thus leaving general practitioners with the task and difficulty of identifying ocular pathological conditions.

In addition, there is irregular distribution of ophthalmology specialists in many countries, which makes it difficult for the population to access adequate eye care.^1^ Within this scenario, artificial intelligence (AI) may offer an alternative that could increase the population’s access to eye care.^2,3^

Formation of a convolutional layer is the basis of an algorithm. This transforms the input data through application of a set of filters to produce a final response (such as the output). Neural networks set weights on their own for the filters used during the training process. The filters are defined before the training phase but can be optimized during the learning process.

During the learning phase, algorithm performance can be improved. This phase can be supervised when data is assigned during training. It can also be unsupervised and, in this case, the device creates its own input sample.

The training and development phase of an algorithm is generally divided into training, validation and test data sets. These data sets should not be repeated: hence, an image that is in one of the data sets (for example, training) should not be used in any of the other data sets (for example, validation). The data set used during the training phase can be made as subsets and can be optimized through retro-propagation of the information collected.

The data set used in validation is used for selection of parameters and adjustments, and for implementation of training conditions. After the training phase, independent test data is used, captured using different devices, from different populations under different clinical contexts.

When examining the performance results from an algorithm, it is important to evaluate the methodology and the way in which it was developed. For example, an algorithm developed for analysis of fundus retinography may perform poorly if applied to a retinal photograph with a larger field.^4–9^

## OBJECTIVE

The purpose of this article was to provide an overview of the basic principles of AI and its main studies in the fields of glaucoma, retinopathy of prematurity, age-related macular degeneration and diabetic retinopathy. From this perspective, the limitations and potential challenges that have accompanied implementation and development of this new technology within ophthalmology are presented.

## METHODS

We searched the literature on the main applications of artificial intelligence within ophthalmology, using the keywords “artificial intelligence”, “diabetic retinopathy”, “macular degeneration age-related”, “glaucoma” and “retinopathy of prematurity”, covering the period from January 1, 2007, to May 3, 2021. We used the Medical Literature Analysis and Retrieval System Online (MEDLINE) database (via PubMed) and the Latin American and Caribbean Literature in Health Sciences (Literatura Latino-Americana e do Caribe em Ciências da Saúde, LILACS) database (via Virtual Health Library) to identify relevant articles.

Through this search, we selected and reviewed articles on the potential automated clinical applications of artificial intelligence technologies and big data analysis. A summary of the articles selected is provided below. The details of the search strategy are shown in Table 1.

**Table 1:** Details of the search strategy

Database	Search strategies	Papers found
MEDLINE (via PubMed)	(“artificial intelligence”) and (“diabetic retinopathy”)	226
MEDLINE (via PubMed)	(“artificial intelligence”) and (“macular degeneration age-related”)	53
MEDLINE (via PubMed)	(“artificial intelligence”) AND (“glaucoma”)	151
MEDLINE (via PubMed)	(“artificial intelligence”) and (“retinopathy of prematurity”)	26
LILACS (via Biblioteca Virtual em Saúde)	(“artificial intelligence”) and (“diabetic retinopathy”)	188
LILACS (via Biblioteca Virtual em Saúde)	(“artificial intelligence”) and (“macular degeneration age-related”)	90
LILACS (via Biblioteca Virtual em Saúde)	(“artificial intelligence”) and (“glaucoma”)	118
LILACS (via Biblioteca Virtual em Saúde)	(“artificial intelligence”) and (“retinopathy of prematurity”)	21

LILACS = Latin American and Caribbean Literature in Health Sciences (Literatura Latino-Americana e do Caribe em Ciências da Saúde); MEDLINE = Medical Literature Analysis and Retrieval System Online.

## RESULTS

**Figure 1:**
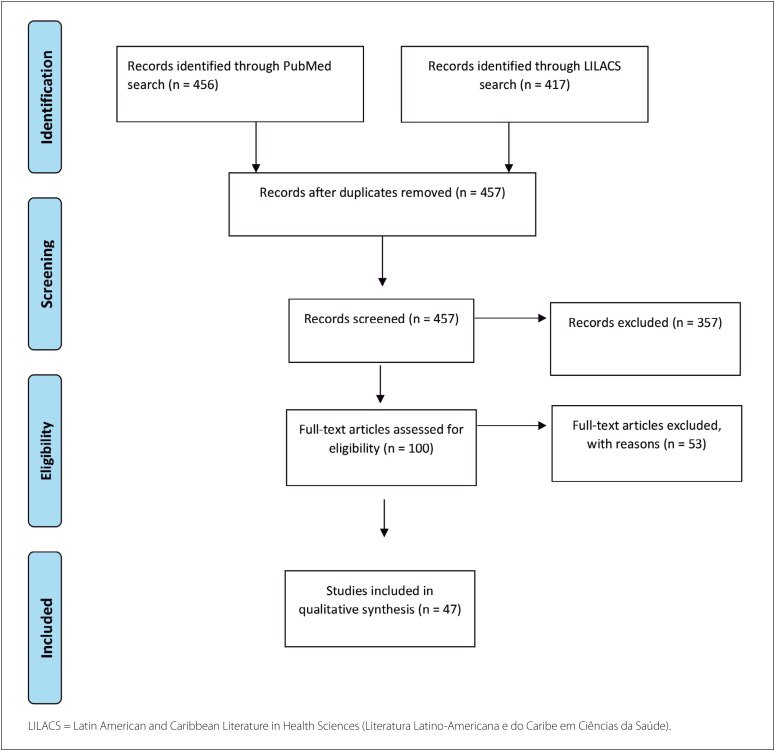
Flow diagram of the study selection process.

From the search in the databases, one clinical trial, four meta-analyses, four randomized controlled trials, 47 reviews and four systematic reviews were identified. After screening the titles and abstracts, removing duplicates and screening the citations, 47 studies were considered eligible for critical analysis. The article selection process is detailed in Figure 1.

### Diabetic retinopathy

Diabetes is the leading cause of blindness in adulthood, affecting more than 415 million people worldwide.^10,11^ Recent studies on the use of AI for monitoring diabetic retinopathy have demonstrated that it has high precision for detecting this disease.^10,12,13^

In 2018, IDx-DR, which is an AI diagnostic system that autonomously diagnoses patients with diabetic retinopathy (including macular edema), was approved by the United States Food and Drug Administration (FDA) for classifying diabetic retinopathy. This was the first artificial intelligence device approved by that institution.

Ting et al.^12^ evaluated the performance of artificial intelligence for screening for diabetic retinopathy, macular degeneration and glaucoma, using 494,661 images of the retina. This algorithm was then tested externally in 11 multiethnic cohorts. For detecting diabetic retinopathy, its sensitivity was 90.5% and specificity was 91.6%. For diabetic retinopathy with the risk of vision loss, its sensitivity was 100% and specificity was 91.1%. For macular degeneration, its sensitivity was 93.2% and specificity was 88.7%. For glaucoma, its sensitivity was 96.4% and specificity was 87.2%.

Tufail et al.^10^ evaluated automated screening of patients with diabetic retinopathy by evaluating systems for automatic detection of diabetic retinopathy in comparison with human graders. EyeArt had sensitivity of 94.7% for detecting diabetic retinopathy and 93.8% for referable retinopathy (maculopathy, proliferative or pre-proliferative diabetic retinopathy). Retmaker had sensitivity of 73% for any retinopathy, 85% for referable retinopathy and 97.9% for proliferative retinopathy. AI models were trained to detect microaneurysms, retinal hemorrhages and hard or soft exudates. Retmaker is a system that has been used for screening diabetic retinopathy.^10^

Abràmoff et al.^14^ evaluated an algorithm for automatic detection of diabetic retinopathy, with specificity of 59.45% and sensitivity of 96.8%. Gulshan et al.^15^ developed an algorithm for screening diabetic retinopathy using 128,175 images of color fundus retinography, which had specificity of 90.3% and sensitivity of 98.1%, and reached an area below the receiver operating characteristic (ROC) curve of 0.99 for detecting referable diabetic retinopathy.

Subsequently, Gulshan et al.^16^ investigated use of an algorithm in 10 primary care centers for six months, which resulted in sensitivity of 87.2% and specificity of 90.8% for detecting clinically significant macular edema in at least one eye. This follow-up study emphasized the importance of testing an artificial intelligence algorithm in the real world.

Li et al.^13^ developed an artificial intelligence-based model for detecting diabetic retinopathy based on the color of retinography photographs. Its sensitivity was 97.0% and specificity was 91.4%, as result of using more than 100,000 images. It reached an area below the ROC curve of 0.99 in validation and 0.955 in external validation using an independent multiethnic data set.

Gargeya et al.^17^ published a study in which 75,137 fundus retinography photos from diabetic patients were used to train and test an artificial intelligence model. The model had sensitivity of 94% and specificity of 98%.

The data should preferably be validated using different camera systems and populations. Some diabetic retinopathy assessment systems only evaluate the central 45 degrees, close to the macula, and do not assess diabetic retinopathy lesions that may be occurring on the periphery of the patient’s retina.^16^

Because of the great variation in the reference standards between different studies, it is difficult to compare the performance of the algorithms. To solve this challenge, algorithms could be tested on an independent data set with a single reference standard.

### Age-related macular degeneration

Diabetic retinopathy and age-related macular degeneration (AMD) are the leading causes of blindness among adults over the age of 50 years in the United States. Just like in relation to diabetic retinopathy, development of algorithms for diagnosing and monitoring AMD has therefore been stimulated. AMD cases normally need to be referred to a tertiary-level eye service for clinical evaluation by experts.^18–20^

### Algorithms evaluating color fundus photos

Ting et al.^12^ used a database of 72,610 fundus retinography images to classify these patients into intermediate and advanced levels of macular degeneration, with sensitivity of 93.2% and specificity of 88.2%.

Burlina et al.^21^ classified patients using software developed from 130,000 images on 4613 patients and reported that it showed 91.6% accuracy for identifying moderate and advanced macular degeneration. The findings from their study resulted in classification of these patients into four stages, ranging from 1 (without signs of macular degeneration) to 4 (advanced stage).

Grassmann et al.^22^ tested an algorithm using 120,656 fundus retinography images from 3,654 patients and reported that it showed accuracy of 84.2% for differentiating between early and late macular degeneration and 94.3% for identifying healthy individuals. These investigators used 13 scales based on the Age-Related Eye Disease Study (AREDS), such that stage one showed no signs of degeneration of the macula, stages two to nine represented intermediate disease and stage ten represented the late stage of the disease.

Peng et al.^23^ evaluated the severity and risk of progression of macular degeneration using fundus color photography. The performance of the algorithm was compared with that of retinal specialists, and it demonstrated accuracy of 0.94 for detecting large drusen, 0.93 for pigmentary abnormalities and 0.97 for advanced AMD.

### Algorithms evaluating optical coherence tomography

Bogunovic et al.^24^ developed a machine learning method for estimating a risk score and biomarkers associated with progression of macular degeneration in optical coherence tomography (OCT) examinations. In addition, Bogunovic et al.^25^ evaluated an algorithm for analyzing OCT images in order to predict the best mode of treatment with intravitreal injection. The main predictive characteristic found in the latter study was the presence of subretinal fluid in the central 3 mm of the macular OCT image.

Schlegl et al.^26^ developed an AI model for detecting and quantifying intra and subretinal fluid. This algorithm performed well in detecting these lesions in patients with macular degeneration and central retinal vein occlusion. The method had accuracy of 0.94 for detecting intraretinal fluid relating to macular degeneration, diabetic macular edema and retinal vein occlusion. The accuracy for detecting subretinal fluid was 0.92, with superior performance among patients with macular degeneration and retinal vein occlusion, compared with patients with macular edema due to diabetes.^27^

Venhuizen et al.^28^ demonstrated the use of AI for classifying the severity of macular degeneration, with sensitivity of 98.2% and specificity of 91.2%. Using a database of 100,000 OCT B-scans (50% from examinations without alteration and 50% from examinations with macular degeneration), Lee et al.^29^ reported that their algorithm showed accuracy of 87.6% with sensitivity of 84.6% and specificity of 91.5%. That algorithm was developed using images from OCT, diagnoses of macular degeneration provided by a specialist and worst vision of 20/30 in the better eye.

Thus, the main algorithms that have been developed are useful for detecting and segmenting injuries, estimating the risk of progression to advanced stages or evaluating the risk of conversion of dry AMD to an exudative form.

### Glaucoma

Glaucoma is an important cause of loss of vision worldwide. In evaluating optic neuropathy, the cup disc needs to be characterized: its size and shape can vary between people. However, defining the cup disc is insufficient for diagnosing glaucoma, due to the large anatomical changes of the optic disc. Examination of the OCT retinal nerve fiber layer thickness and ganglion cell complex can be used for diagnosing glaucoma. Visual field examination is inexpensive and can be used to assess functional loss. However, the sensitivity and specificity of the diagnosis is lower than when a combination of visual field and OCT data is used.

Use of anatomical and functional data together is superior to anatomical data in isolation for diagnosing glaucoma. Artificial intelligence algorithms can combine these factors to aid in making diagnoses. In a study using a database of 125,189 fundus retinography images, Ting et al.^12^ reported that their algorithm had sensitivity of 96.4% and specificity of 87.2% for detecting suspected glaucoma, defined as discs with an upper excavation of 0.8 and/or glaucomatous changes. Li et al.^30^ evaluated a machine learning algorithm for detecting glaucoma based on 48,116 color fundus photographs, with sensitivity of 95.6%, specificity of 92% and an area under the ROC curve of 0.986. The main cause of false negatives in their study was patients with high myopia. Physiological excavation of the optic disc was the most common cause of false positives in their study. A study using deep learning to classify suspected glaucoma using OCT examinations was developed to differentiate healthy eyes from initial glaucoma.^31^

Kim et al.^32^ developed a model based on machine learning using three types of records including retinal nerve fiber layer thickness, visual field and ophthalmic clinical data. These investigators reported accuracy of 0.98, sensitivity of 0.983 and specificity of 0.975.

Ahn et al.^33^ developed an algorithm that only required retinography data. Use of this limited data resulted in accuracy of 92.2% for identifying glaucoma cases. This model may be convenient with regard to helping with screening of glaucoma cases. Asaoka et al.^34^ used artificial intelligence to analyze visual fields in patients with pre-perimetric open glaucoma and were able to differentiate them from patients with healthy eyes, with good accuracy (92.6%).

Using 3242 retinography images from eyes with confirmed glaucoma, Shibata et al.^35^ developed a trained and tested algorithm, from which they reported an excellent area under the ROC curve, of 0.965. This algorithm was trained to detect cup size, optic disc notch, nerve fiber layer atrophy, peripapillary atrophy and optic disc hemorrhage.

Masumoto et al.^36^ used 1,379 retinography images to detect glaucoma, and found 80.2% specificity and 81.3% sensitivity. The values were higher for severe cases of glaucoma.

Elze et al.^37^ developed an AI system for identifying patterns of glaucomatous and non-glaucomatous visual field (VF) loss. Through an analysis on 13,231 reliable Humphrey VFs, they identified an ideal solution with 17 prototypes of glaucomatous vision loss. Algorithms show great difficulty in detecting the early stages of glaucoma when patients do not have defects in the visual field. Thus, studies using longitudinal data are needed in order to correctly identify patients who will develop glaucoma.

In patients with severe glaucoma, disease identification by means of algorithms usually has better results. However, caution needs to be exercised due to the great anatomical variability of optic nerves in populations, especially among patients with a high degree of myopia.

### Retinopathy of prematurity

Retinopathy of prematurity (ROP), which has a prevalence of 6%-18%, is one of the main causes of loss of vision in childhood worldwide.^38^ This disease, in its third epidemic, resulted in irreversible blindness in more than 50,000 premature newborns because of a shortage of trained specialists.^39,40^

Experts usually disagree about the clinical classification of ROP. In the cryotherapy (CRYO)-ROP study, the second examiner disagreed with the first regarding the diagnosis of threshold disease in 12% of the cases.^41^ Also, in a multicenter telemedicine study on diagnosing ROP, almost 25% of the tests did not align with one of the three criteria for clinically significant ROP.^42^

The initial approaches to automated image analysis have been based on quantification of vascular tortuosity and vascular dilation. These systems were developed and validated for wide-angle RetCam images. They were evaluated based on the diagnoses of specialists but did not have any application in the real world because they are only semi-automated, thus requiring manual identification.^43^

The initial computational approaches for detecting this pathological condition focused on the vascular tortuosity of retinopathy of prematurity-plus (ROP-plus).^44^ Recent work has suggested other possibilities for assessing vessel angles as resources for predictive values for this disease, using linear logistic regression models.^45^

Brown et al. developed and validated a fully automated deep learning system called informatics-retinopathy of prematurity deep learning (i-ROP DL), using a database of 5,511 retinography images obtained by means of a RetCam background camera. This enabled diagnosis of three levels of ROP (plus, pre-plus and normal), with an area under the ROC curve of 0.98 for a positive diagnosis of the disease, in comparison with a reference standard defined by specialists. The i-ROP DL system reached specificity of 94% and sensitivity of 93% for diagnosing ROP-plus and 94% specificity and 100% sensitivity for diagnosing ROP at pre-plus or worse levels.^46^

In an algorithm developed by Redd et al.,^47^ an area under the ROC curve of 0.96 was found for identifying type 1 ROP and 0.91 for clinically significant ROP.^47^ Xiao et al. developed an AI program that quantified the area of neovascularization in patients with ROP.^48^ The AI program reached a higher range of correlation coefficients than that of specialists, for classification of areas with neovascularization. The algorithm works for quantification of key values of oxygen-induced retinopathy images, using deep learning neural networks. Ataer-Cansizoglu et al. developed an AI program with 95% accuracy for analyzing vascularization data.^49^

Current methods for detecting ROP can distinguish between mild and severe cases of ROP but are still unable to identify the stage of the disease.^50^ Campbell et al.^51^ demonstrated that automated diagnosis of ROP (i-ROP) had an accuracy of 95%, while the average accuracy of 11 specialists was 87%. Thus, algorithms with performance comparable to that of retinal specialists already exist.

## DISCUSSION

Development of algorithms for diagnosing ophthalmic diseases requires many images in order to achieve a classification. When an algorithm is designed, the following need to be considered: the population in which it will be applied, whether it is aligned with current clinical evidence and whether use of the algorithm applies only to diagnosing the disease.^52^

Because diseases such as glaucoma, macular degeneration, diabetic retinopathy and retinopathy of prematurity have relatively high prevalence, this favors creation of algorithms, given the large amount of data that has been documented. Rare diseases, with limited data, still present a challenge with regard to development of artificial intelligence programs. Among the topics selected for the present review, retinopathy of prematurity is one for which the fewest algorithms have been developed. This is thought to be due to the lower prevalence of this pathological condition in relation to the others analyzed and the greater difficulty in documenting data among preterm patients. Development of new portable devices that document retinopathy of prematurity may contribute towards development of new algorithms in the future.

Ethical and legal aspects should always be considered by groups that develop algorithms, with the aims of avoiding racial prejudice in healthcare and preserving fundamental rights to protection of personal data.

Currently, there are large databases (big data) of electronic medical records and digital images, which enable recognition of patterns in large volumes of data within a short period of time, thereby reducing errors in diagnostics and therapeutics and creating personalized medicine. In this context, a large database called Intelligent Research in Sight was created to store data on 17,363,018 patients from 7200 ophthalmologists in the United States, in order to improve individual care and public policies.^53^

The data of some algorithms can be used for three major groups of purposes: classification, segmentation and prediction. In classification, an image will be classified in different categories (presence or absence of disease, for example). This function is explored in disease screening and staging algorithms. In segmentation algorithms, different anatomical structures and lesions of importance in determining disease biomarkers are outlined. Prediction algorithms, on the other hand, address the relationship of data with future results, and thus help in estimating disease prognosis.

Some AI programs have multiple layers of information input and output, thus enabling a more efficient machine learning process that not only classifies the parameters, but also extracts the results. Most metrics within performance analysis have included calculation of sensitivity, specificity and the area under the ROC curve, which is calculated from sensitivity and specificity values. The closer to 1.0 that the area under the ROC curve reaches, the greater the sensitivity/specificity of the method is. Moreover, it is necessary to evaluate the sensitivity under a fixed specificity. An artificial intelligence system with a good area under the ROC curve may have low sensitivity at a high level of specificity, thus resulting in a high rate of false negatives (Table 2).^10,12,14–16,21–23,28–30,32–34,36,46,49,51^

**Table 2:** Comparison of accuracy, sensitivity, specificity and number and type of images analyzed

Authors	Pathological condition/ number of images analyzed	Precision
Ting et al.^12^	Diabetic retinopathy/76,370 images of retinal photographs	Sensitivity of 90.5% and specificity of 91.6%
Tufail et al.^10^	Diabetic retinopathy/20,258 images of retinal photographs	EyeArt (sensitivity of 93.8%) and Retmaker (sensitivity of 97.9%)
Abràmoff et al.^14^	Diabetic retinopathy/1,748 images of retinal photographs	Sensitivity of 96.8% and specificity of 59.4%
Gulshan et al.^15^	Diabetic retinopathy/9,963 images of retinal photographs	Sensitivity of 98.1% and specificity of 90.3%
Gulshan et al.^16^	Diabetic retinopathy/103,634 images of retinal photographs	Sensitivity of 87.2% and specificity of 90.8%
Ting et al.^12^	AMD/72,610 images of retinal photographs	Sensitivity of 93.2% and specificity of 88.2%
Burlina et al.^21^	AMD/130,000 images of retinal photographs	91.6% accuracy
Grassmann et al.^22^	AMD/120,656 images of retinal photographs	84.2% accuracy
Venhuizen et al.^28^	AMD/3,265 images of OCT	Sensitivity of 98.2% and specificity of 91.2%
Peng et al.^23^	AMD/58,402 images of retinal photographs	Accuracy of 97.0%
Lee et al.^29^	AMD/48,312 images of OCT	Sensitivity of 84.6% and specificity of 91.5%
Ting et al.^12^	Glaucoma/125,189 images of retinal photographs	96.4% sensitivity and 87.2% specificity
Li et al.^30^	Glaucoma/48,116 images of retinal photographs	Sensitivity of 95.6% and specificity of 92%
Kim et al.^32^	Glaucoma/399 images of visual field	Sensitivity of 98.3% and specificity of 97.5%
Ahn et al.^33^	Glaucoma/1,542 images of retinal photographs	92.2% accuracy
Asaoka et al.^34^	Glaucoma/171 images of visual field	92.6% accuracy
Masumoto et al.^36^	Glaucoma/982 images of visual field	Sensitivity of 81.3% and specificity of 80.2%
Brown et al.^46^	ROP/5,511 images of retinal photographs	93% sensitivity and 94% specificity
Ataer-Cansizoglu et al.^49^	ROP/77 images of retinal photographs	95% accuracy
Campbell et al.^51^	ROP/77 images of retinal photographs	95% accuracy

AMD = age-related macular degeneration; OCT = optical coherence tomography; ROP = retinopathy of prematurity.

It is important to highlight the reliability of the ground truth labels, which in ophthalmological studies are evaluations by specialists, who may nevertheless have divergent opinions. It is important that the sample used for training the algorithms should be specified.

Incorporation of machine learning technology within ophthalmology can improve medical care for the population in regions with limited medical resources, thus reducing some social inequalities.

### Future directions, strengths and limitations

Development of enormous longitudinal studies to judge the artificial intelligence systems developed is important for assessing the real security and effectiveness of artificial intelligence systems. Narrative reviews contribute towards providing updates on the medical knowledge available. These reviews help in formulating new research projects based on interpretation of the results from published studies after non-systematic analysis. New studies that use algorithms that combine analysis on OCT images, fundus retinography and visual fields can be useful for diagnosing and evaluating multiple pathological conditions simultaneously.^12^

Development of algorithms with real-time cloud information analysis is often helpful within the management and monitoring of eye diseases. However. studies need to be carried out with the aim of preventing certain decisions made by algorithms from going beyond certain ethical and moral precepts that have been established by society. Biases in data collection can substantially affect the generalization of the trained model beyond the population in which it was trained.

Thus, further studies are needed, with algorithms developed in different populations. The data used for external validation should come from a geographically distinct population, with validation by independent researchers.^54^ Studies should be developed with protocols that promote transparency in clinical trials, in order to validate the use of algorithms.^55^

The current algorithms have been developed to evaluate two-dimensional images. Incorporation of multimodal images in the training of the algorithms can facilitate identification of three-dimensional ocular pathological conditions.

The articles included in this review generated heterogeneous data because of the diversity in the design of the studies. The main limitation of this review was the lack of tools for methodological assessment of the reviews. In addition, this narrative review does not provide quantitative answers to specific questions about studying artificial intelligence.

## CONCLUSION

Use of technology, as embodied in artificial intelligence algorithms, is a way of providing an increasingly accurate service and enhancing scientific research. This forms a source of complement and innovation in relation to the daily skills of ophthalmologists. Thus, artificial intelligence adds technology to human expertise.
